# Can Labs Help With Vaccination? In Vitro Tests in Diagnosis of Allergy to COVID‐19 Vaccines–A Systematic Review

**DOI:** 10.1002/iid3.70206

**Published:** 2025-05-14

**Authors:** Jan Romantowski, Marika Gawinowska, Piotr Trzonkowski, Marek Niedoszytko

**Affiliations:** ^1^ Department of Allergology Medical University of Gdansk Gdansk Poland; ^2^ Department of Immunology Medical University of Gdansk Poland

**Keywords:** basophile activation test, ELISA, hypersensitivity, IgE, laboratory, lymphocyte activation test, vaccine

## Abstract

**Introduction:**

Since the outbreak of the coronavirus pandemic in 2019, vaccinations have proven to be a key strategy in disease prophylaxis. Although vaccines are safe from the perspective of the general population, hypersensitivity reactions have still been described, causing individuals to be reluctant in their vaccination decision. Since the description of first reports of COVID‐19 vaccine allergy, many protocols of allergy work‐up have been developed, including In Vitro and In Vivo tests. Although In Vivo tests were more accessible, many patients preferred In Vitro tests that would not involve contact with the allergen and be safe. This applied in particular to patients that had experienced a severe delayed hypersensitivity reaction in which In Vivo tests were highly limited and provocations were deemed high risk. Taking into account these circumstances, In Vitro tests might significantly enhance allergy work‐up.

**Methods:**

National Center for Biotechnology Information (Pubmed) database was searched in May 2024 for articles on In Vitro diagnostic methods for COVID‐19 vaccine allergy and hypersensitivity.

**Results:**

This article describes the In Vitro tests developed to date in the diagnosis of COVID‐19 vaccine hypersensitivity: (1) analysis of specific IgE and IgG, (2) Basophil Activation Test, (3) Histamine Release Test, (4) IgM‐dependent complement activation, (5) Lymphocyte Transformation Test, (6) Flow cytometry T‐Cell markers, (7) Th1/Th2 cytokines concentration in cell culture.

**Conclusions:**

The article highlights the tests' advantages, flaws and possible clinical applications.

## Graphical abstract

The article describes novel approach to COVID‐19 allergy diagnosis protocols. To date most of them revolved around In Vivo tests (skin testing, provocations). We describe and propose implementation of In Vitro tests in allergy work‐up for those patients.

## 1 Introduction

The COVID‐19 pandemic highlighted new challenges for modern medicine [1, 2]. With limited effective treatment options, infection prophylaxis was the most important strategy in disease control. One year after the outbreak, new vaccines were developed, and massive population‐wide vaccination campaigns were initiated [3]. At that point, reports of allergic reactions to vaccines and/or their excipients prompted the need for allergy work‐up methods to determine possible contraindications or to enable personalized choice of a vaccine regimen in particular patient administrations [4, 5, 6].

Meanwhile, multiple diagnostic tools towards COVID‐19 vaccine allergy have been proposed [5, 7, 8, 9]. These include In Vivo procedures: (1) Skin Prick Tests (SPT), (2) Intradermal Tests (IDT), (3) Patch Tests, (4) Provocations and challenges. Additionally, In Vitro tests have been developed: (1) specific IgE and IgG, (2) Basophil Activation Test (BAT), (3) Histamine Release Test (HRT), (4) IgM‐dependent complement activation, (5) Lymphocyte Transformation Test (LTT), (6) Flow cytometry T‐Cell markers, (7) Th1/Th2 cytokines concentration in cell culture. A general comparison between the In Vitro and In Vivo tests is presented in Table 1.

**Table 1:** Comparison of utility between In Vivo and In Vitro tests in COVID‐19 vaccine hypersensitivity diagnostics [7, 8, 10].

	In Vivo	In Vitro
Accessibility	Easier to perform, usually no additional equipment required	Easy blood collection, but requires access to advanced standard laboratory equipment
Reliability	High in provocations/challenges, high in immediate hypersensitivity, probably low/difficult to assess in delayed hypersensitivity	Strongly depends on the type of reaction, relatively high in delayed hypersensitivity
Time to result	Fast, results within few hours (with the exception of patch tests). Provocations take a few days.	Usually from a few hours to up to a week
Cost per patient	Low, but requires trained allergology personnel	High
Safety	Risk of allergic reactions on exposure during tests and provocations/challenges	No significant risks associated

Both In Vitro and In Vivo had their use in allergy work‐ups before COVID‐19 vaccinations and it is imperative to personalize the test according to the main symptoms in the patient interview. The relatively high costs of In Vitro tests probably make them unable to be performed in large numbers of patients, thus diagnosing only selected cases. On the other hand, the lower safety of In Vivo tests usually restricts them to specialized allergology hospital departments. It must be noted that laboratory procedures sometimes require removing standard wash buffers that might contain polysorbate [11].

## 2 Methodology

National Center for Biotechnology Information (Pubmed) database was searched in May 2024 by two study team members for articles on In Vitro diagnostic methods for COVID‐19 vaccine allergy and hypersensitivity. “COVID‐19 vaccine allergy test” key words revealed 450 articles. These were manually reviewed in context of vitro tests such as “BAT,” “specific IgE.” We identified 15 studies in this narrow area, with most of them series of cases type. Judging by low amount of scientific reports all articles were included in the analysis. Article by Barbaud et al. was used as main direction for work‐up protocols. Article has been prepared using PRISMA 2020 guidelines [12].

## 3 Clinical Phenotypes of Vaccine Hypersensitivity

Although COVID‐19 vaccines proved to be safe in population‐wide administrations, hypersensitivity reactions have still been described. The most typical are side effects, often called type A reactions [13]. They usually last 1–2 days, are mild and do not require medical healthcare attention [14]. The most typical include fatigue, muscle and joint pain, injection site reactions, headache, and fever. Injection site reactions were reported by one‐third of patients and significant systemic symptoms varied from around 4% for dyspnea to 40% for fatigue. The occurrence of side effects seems consistent throughout the second and booster doses [15]. Some of the local reactions result from the route of administration, and general ones such as fatigue or fever might result from proper immune system activation with a type I IFN increase [16]. Thus, those reports are usually not considered a contraindication for further administrations.

Drug allergy manifestations are usually divided into two groups based on their time of occurrence: (1) immediate—usually start < 24 h after exposure, and (2) delayed—may start after a few hours, although vast majority occur 24 h or even up to 3 months postadministration [17]. Immediate reactions typically include type I hypersensitivity, and their symptoms include, for example, urticaria, angioedema, bronchospasm, anaphylaxis, diarrhea, and rhinitis [18]. Delayed reactions, with prevalent type IV mechanisms, include urticaria, maculopapular rash, dermatitis, Stevens‐Johnsson Syndrome, Drug Reaction with Eosinophilia and Systemic Symptoms, acute generalized exanthematous pustulosis. Due to the different mechanisms, including the variety of cytokines and antibodies that lead to those manifestations, different, personalized tests should be applied. In this circumstance, vaccination is recommended only after an allergy work‐up [9]

Some microbial infections might induce autoimmunity based on molecular mimicry between infection and self‐antigens [19, 20, 21, 22]. Similarly, vaccination that delivers antigens for immunity development might produce autoimmune disorders, though the cause‐effect is usually uncertain, and an argument of random coincidence remains. It also is important to point out that some autoimmune disorders such as chronic spontaneous urticaria at early stages might present an identical clinical picture as allergic urticaria [21]. Thus, longer observation is required for a final diagnosis. An allergy work‐up in this patient group would probably not reveal hypersensitivity results.

## 4 Testing With Excipient Versus Vaccine

The main cause of allergic reactions to COVID‐19 vaccines is considered excipients. These are listed in Table 2. According to Barbaud et al., if the patient experienced a significant or severe allergic reaction after receiving a COVID‐19 vaccine, diagnostic tests should be performed primarily with the vaccine, and excipient tests may be considered. If the patient has a history of allergic reactions post other injectable drugs/vaccines, the diagnostic process should start with excipients and, in the case of a positive result, careful COVID‐19 vaccine selection and administration in an Allergy Unit. All mild reactions might proceed with vaccinations without testing, though individual observation is suggested [9].

**Table 2:** List of key excipients in COVID‐19 vaccines and their other sources that could help in the patient interview or planning allergy work‐up [9].

Excipient	COVID‐19 vaccine	Examples of other sources
Polysorbate 80	Nuvaxovid, Janssen, AstraZeneca, Sputnik V	Soaps, cosmetics, shampoos
PEG2000	Comirnaty, Spikevax	Laxatives, cosmetic creams
Tromethamine	Spikevax, Sputnik V	Cosmetic creams
Polysorbate 20	Sanofi	Cosmetics, food products
EDTA	AstraZeneca	Textiles, paper, eyedrops, food

The question of testing with the excipient and/or vaccine remains open. In some cases, particularly, antibodies IgE and IgG, only excipient tests are available. The excipient tests give the patient knowledge about other possible drug hypersensitivities. On the other hand, vaccine tests predict the sensitivity to the exact regimen that might be administered to the patient immediately after the diagnostic process is completed, and also provide information about sensitivity to other substances in the vaccine, not only those most prevalently allergising, such as PEG or Polysorbate [5, 9]. In other words, vaccine tests provide complete results regarding this particular regimen, while excipient tests also provide results on other drug administrations in the future. The choice should be personalized based on the patient interview and previous vaccinations.

## 5 Excipient‐Specific IgE and IgG

The role of allergen‐specific IgE has been thoroughly described in immediate, type I hypersensitivity reactions [18]. The presence of specific IgE together with a positive interview usually confirms allergy with a relatively high risk in drug‐related reactions. The role of IgG remains unclear. It has been proven to either induce tolerance or have no effects in some allergens, such as food [23]. However, IgG in some situations might activate mast cells, causing pseudo‐allergic reactions [24]. Detection of specific immunoglobulins in allergology is usually done with the ELISA test with the excipient on a plate exposed to the patient's serum. Mouri et al. evaluated PEG‐specific IgE, PEG‐specific IgG, Polysorbate‐specific IgE and SPT in patients with an allergy to COVID‐19 vaccines [25]. In the results, significantly higher specific IgE and IgG were observed in the patient group as well as a positive SPT. The authors highlight the utility of both immunoglobulin classes in the diagnosis of vaccine excipient allergy before vaccination, though they admit that those results might be false positives in the healthy, nonallergic population. Mortz et al. suggested a complete allergy work‐up protocol using SPT, Basophil Histamine Release Test and excipient‐specific IgE with patients already diagnosed with PEG allergy [11]. In an evaluation of 23 patients, two had a positive SPT with the vaccine and its respective excipient. The reproducibility of the SPT after 2–6 years was as low as 40%. Of those nine SPT‐positive patients, only four had positive PEG 2000/PEG 10,000‐specific IgE. This leads to the conclusion that both SPT and IgE decrease relatively quickly in patients with confirmed PEG allergy. It is also worth mentioning that IgG may play both an allergenic and tolerogenic role in COVID‐19 vaccine hypersensitivity. In addition, the presence of specific IgG may interfere with the binding of the same antigens to specific IgE, thus lowering the sensitivity of the IgE results [26].

## 6 Basophil Activation Test

The BAT is an In Vitro diagnostic tool used primarily in the assessment of drug allergies, particularly those involving immediate hypersensitivity reactions [27]. The methodology involves incubating a blood sample containing live cells from the patient with a potential allergen, along with both a negative and a positive control (IgE‐dependent and IgE‐independent stimuli). Subsequently, the expression of basophil activation markers on their surface, which occurs in response to contact with the tested agent, is evaluated using flow cytometry and monoclonal antibodies labeled with fluorochromes. The most commonly used basophil activation markers are CD63 and CD203c. The test results are presented as the percentage of activated basophils. Additionally, the stimulation index (SI) is used, which is the ratio of activated basophils after allergen stimulation compared to nonstimulated basophils (negative control).

When evaluating BAT results, it is important to consider certain limitations. It is estimated that approximately 5%–15% of the tested population may temporarily be nonresponders [27, 28]. In this group, basophils do not activate in the positive control (IgE‐dependent, both in terms of CD63 and CD203c). As a result, it is not possible to reliably assess the outcome with the allergen, including the COVID‐19 vaccine. Furthermore, attention is drawn to cellular anergy, which can be temporary due to recent exposure to the allergen, or permanent, resulting from the time elapsed since the last contact with the allergen and the reduction in specific IgE levels [27]. Notably, the immune response triggered by the COVID‐19 vaccine does not affect basophil reactivity [29]. However, having had COVID‐19 may lead to positive BAT results with a drug, as observed in the control group of patients without a drug allergy in Labella's study [29]. Although this observation was not confirmed in two subsequent studies [30, 31], it is important to consider recent SARS‐CoV‐2 infection when interpreting BAT results.

There is ongoing discussion regarding the scope of testing: whether to perform BAT with individual vaccine components (such as purified PEG of different molecular weights) or with more complex forms such as PEGylated liposomal nanoparticles, including vaccines. In Li's study, no significant difference in BAT reactivity using pure PEG compared to the mRNA vaccine (BNT162b) was observed [32]. Interestingly, this study demonstrated higher utility for the CD63 activation marker compared to CD203c. Troelnikow described a group of individuals allergic to PEG who reacted to the mRNA vaccine BNT162b2 but not to the traditional form of PEG, both in skin tests and BAT [33]. Subsequent studies, including the largest to date, a multicentre observational study conducted by Pignatii involving 89 individuals with hypersensitivity after vaccination, further emphasize the dominant role of the modified high‐molecular‐weight lipid form of PEG [28, 34, 35]. The exposure of PEG on the surface of lipid nanoparticles undoubtedly increases its allergenicity and improves the sensitivity of the allergologic work‐up.

Overall, many researchers evaluate BAT in the diagnosis of hypersensitivity to PEG and vaccines as a method with high specificity but relatively low sensitivity [28, 30, 32]. A detailed BAT protocol using the mRNA vaccine in patients with PEG allergy, where the sensitivity and specificity of the test were assessed at 60% and 100%, respectively, can be found in Eberlein's study [30]. It may be a useful tool for ruling out PEG allergy due to its high negative predictive value [29]. A limitation of BAT studies, typical for rare allergies such as PEG and vaccine allergies, is the small size of the studied groups.

As mentioned earlier, BAT is most commonly used in the diagnosis of immediate hypersensitivity reactions. However, there are now reports highlighting the role of basophils in delayed reactions and their potential use in allergological diagnostics [34]. Although studies on this topic currently yield conflicting results [36].

## 7 Histamine‐Release Test

The HRT is an In Vitro method for assessing basophil and mast cell activation in response to specific allergens. The test is performed on a freshly collected blood sample. The amount of histamine released during a potential allergic reaction is measured in the supernatant using enzyme immunoassay, radioimmunoassay, fluorometry, or chromatography. Similar to BAT, the test should be performed using only soluble drugs, at appropriate concentrations, and free of cytotoxic potential. The test should be conducted within a few months of the hypersensitivity reaction and allergen exposure [37]. Otherwise, the cellular reactivity measured by histamine release may diminish, even though the skin reactivity in the SPT remains. As mentioned in the BAT section, part of the population does not respond to IgE‐dependent stimuli and is thus termed “nonreleasers.”

There are significantly fewer studies utilizing HRT in the diagnosis of vaccine hypersensitivity and their components compared to BAT. In two studies evaluating HRT in patients with hypersensitivity to PEG or polysorbate, this test was found to be valuable in allergological assessment but less useful than skin tests [37, 38].

## 8 Specific IgM/IgG and Complement System Activation

Activation of the complement system might result from multiple factors. C3a, C4a and C5a are often called anaphylatoxin, because their inflammatory effects mimic classical allergic reactions, including anaphylaxis, in which they can also take part [39]. A study on an animal model showed that PEG‐specific IgM might activate the classical component pathway with sC5b‐9 causing an anaphylactoid reaction [40].

Lim et al. presented a series of three cases of patients who developed erythema, urticaria, edema and bronchospasm post Comirnaty administration [26]. Two of those patients presented increased PEG and vaccine‐specific IgG and IgM together with anaphylatoxin C3a. In these cases, Th2 cytokines IL‐4, IL‐33, and IL‐10 were low. A third patient, however, showed even higher antibody levels, together with high Th2 cytokines and a low C3a. This comparison might suggest that there are possibly two mechanisms: (1) typical allergic observed in the third patient, and (2) pseudo‐allergic observed in the first two cases. This might also explain the negative SPT and ELISA IgE test results in some patients with clear clinical allergic manifestations. In this circumstance, assessment of C3a and cytokines might be of more value than specific IgE. However, diagnostic pre‐vaccination protocols are yet to be developed.

## 9 Lymphocyte Transformation Test

Lymphocyte Transformation Test (LTT) is one of the most common tests used in the diagnosis of delayed hypersensitivity [41]. In this test, peripheral blood mononuclear cells (PBMCs) are sampled from the patient and cultured for 6–7 days together with the suspected drug, a negative control and a positive control—usually lipopolysaccharide. T cell proliferation upon activation is measured with ^3^H‐thymidine uptake during DNA synthesis. Similarly to other culture‐oriented tests, the result is presented as a Stimulation Index, which shows a drug effect related to negative control. The cut‐off points depend on the tested drug and vary from 2 to 4.

Weir et al. presented a description of two patients that experienced delayed hypersensitivity reactions post vaccination and went through an LTT‐oriented allergy work‐up [42]. In the first case, initial administration of Vaxzevria (AstraZeneca) resulted in hepatitis together with thrombocytopenia and leukopaenia. The LTT with Vaxzevria was positive (SI = 6.3) and for Comirnaty, it was negative (SI = 1.6). Thus, an allergy to Vaxzevria was diagnosed, and the patient received Comirnaty with no sequelae. The second patient developed generalized morbilliform eruption 10 days post Comirnaty vaccination. The LTT performed after 3 months revealed high reactivity (SI = 2.0) to Comirnaty but a negative result for Vaxzevria (SI = 0.9). That patient was not vaccinated though, and after a mild COVID‐19 infection, the LTT was repeated with Vaxzevria and Novavax. Interestingly, the results were positive for both vaccines (SI 2.5 and 19.8, respectively) which were never administered to the patient. Finally, the patient was vaccinated with Novavax with no sequelae. Unfortunately, the authors do not describe specifically how the decision was made.

All in all, LTT might prove useful in allergy diagnostic protocols before vaccination; however, the results have to be carefully interpreted for vaccines that were not administered to patient previously. In those cases, false‐positive results are possible.

## 10 Flow Cytometry and Activation Markers

Another way of measuring T‐cell response In Vitro post allergen exposure is measurement of their specific activation markers on the cell surface [41, 43]. A variety of markers have been used to date: CD25, CD69, CD71, CD40L. The markers appear on activated T‐cells and can be measured after 48–72 h on cells cultured in the presence of the tested drug. Additionally, negative and positive controls are performed and the Stimulation Index is usually calculated as the final outcome. The results are usually consistent with LTT, though due to the less frequently use drug test concentration are not well validated. However, the time to result is superior compared to LTT and the specificity/sensitivity seems similar.

In a study on seven patients with severe delayed drug allergies in the past, CD69 and CD40L were performed after 48 h of culture with a COVID‐19 vaccine [36]. Those markers were able to identify two allergic patients with a key impact on the vaccination decision. Both markers also correlated with each other. Due to fast result, cytometry markers seem promising in the diagnosis of delayed allergy to COVID‐19 vaccines.

## 11 Cytokine Concentration in Cell Culture

Another option for measuring the cell activation is measurement of the cytokines that are released [41, 44, 45]. This requires T cell incubation for 48–72 h in the presence of the allergen, a positive and a negative control. The cytokine concentration is usually measured in supernatant using immunoassays, intracellularly with flow cytometry or with gene expression and mRNA assessment.

The choice of cytokines might be difficult due to their nonspecific nature. Usually, a set includes: (1) Th1: IL‐2, IL‐6, IFN‐γ, and TNF‐α; (2) Th2: IL‐4, IL‐5, IL‐10, and IL‐13. The authors usually choose several cytokines to increase the sensitivity and cover both Th1‐ and Th2‐dependent mechanisms [45, 46].

A study by Romantowski et al. measured serum cytokines (IL‐2, IL‐4, IL‐6, IL‐10, IFN‐γ, TNF‐α) together with intracellular cytokine markers (granulysin and INFgamma) in a 72‐h culture with the tested vaccine and PEG [36]. In the results, however, the extracellular cytokines index was greatly increased up to 4000 in both the patients and the healthy controls. The index results were not consistent with other tests, such as the CD69 cytometry marker or BAT. On the other hand, PEG did not show such activation. The authors suggest that the vaccine, being a highly immune‐stimulating agent, increases the cytokine level as its expected drug mechanism. Thus, it might be impossible to differentiate a healthy response from hypersensitivity.

The intracellular markers returned a high index as well, though the results were not consistent with the positive control, which suggests that these might be difficult to perform technically.

## 12 Head‐to‐Head Comparison of In Vitro Methods

Comparison of methods presented above is difficult, mainly due to low number of studies and number of patient participants. This is due to the fact that COVID‐19 vaccines allergies are rare, massive vaccinations are still relatively new and, in some severe cases, thorough diagnostics might be dangerous. Comparison of immediate allergy diagnostic methods are presented in Table 3. Delayed allergy diagnostics is still in early development with only 9 patients evaluated and published to date.

**Table 3:** Comparison of different In Vitro allergy diagnostic methods for immediate hypersensitivity reactions [11, 25, 26, 30, 47, 48, 49, 50, 51, 52, 53].

	Specific IgE/IgG	HRT	BAT	Specific IgM/IgG and complement diagnostics
Excipient testing	Yes	Yes	Yes	Yes
Vaccine testing	No^a^	No^a^	Yes (mainly BNT162b)	Yes
Number of subjects tested	23^b^	35	159^c^	3
Number of positive results (%)	5 (21%)^b^	8 (23%)	62^c^ (39%)	1 (33%)
Correlation with skin tests	Yes	Inconclusive/not tested	Yes (observed in certain studies)	No (different mechanism)

^a^ To date.

^b^ Based only on study by Mortz et al. Mouri et al. did not use cut‐off points, and focused solely on quantitative correlations.

^c^ In one study, immediate and delayed reactions were evaluated together in one group [34].

As presented in Table 3, most studies on immediate hypersensitivity have been performed using BAT and specific IgE/IgG. In these tests, correlation has been observed with SPT and IDT, thus they might prove useful addition in clinical evaluation.

## 13 Allergy Work‐Up Protocols

In patient management, it is important to acquire all information regarding allergic history and previous vaccinations. Barbaud et al. suggest that an allergy work‐up should be performed in patients experiencing immediate or severe allergic reactions to drugs containing the suspected excipient (e.g., PEG), history of immediate or severe reaction to any COVID‐19 vaccine and history of anaphylaxis of unknown cause that might have been associated with the suspected excipient [9]. Local side effects and known systemic side effects are also not considered indications for a diagnostic process, as the tests would not predict them, and the effects are unlikely to be avoided in future administrations.

In the case of immediate reactions, In Vivo skin prick tests and intradermal tests are commonly used and considered standard [9, 54]. HRT and BAT could also be done with a vaccine and/or excipient. Excipient‐specific antibodies in the IgE, IgG, IgM class are mostly useful with excipient allergy. In Vitro tests have uncertain power, though may represent an alternative for patients with contraindications for skin tests.

**Figure 1:**
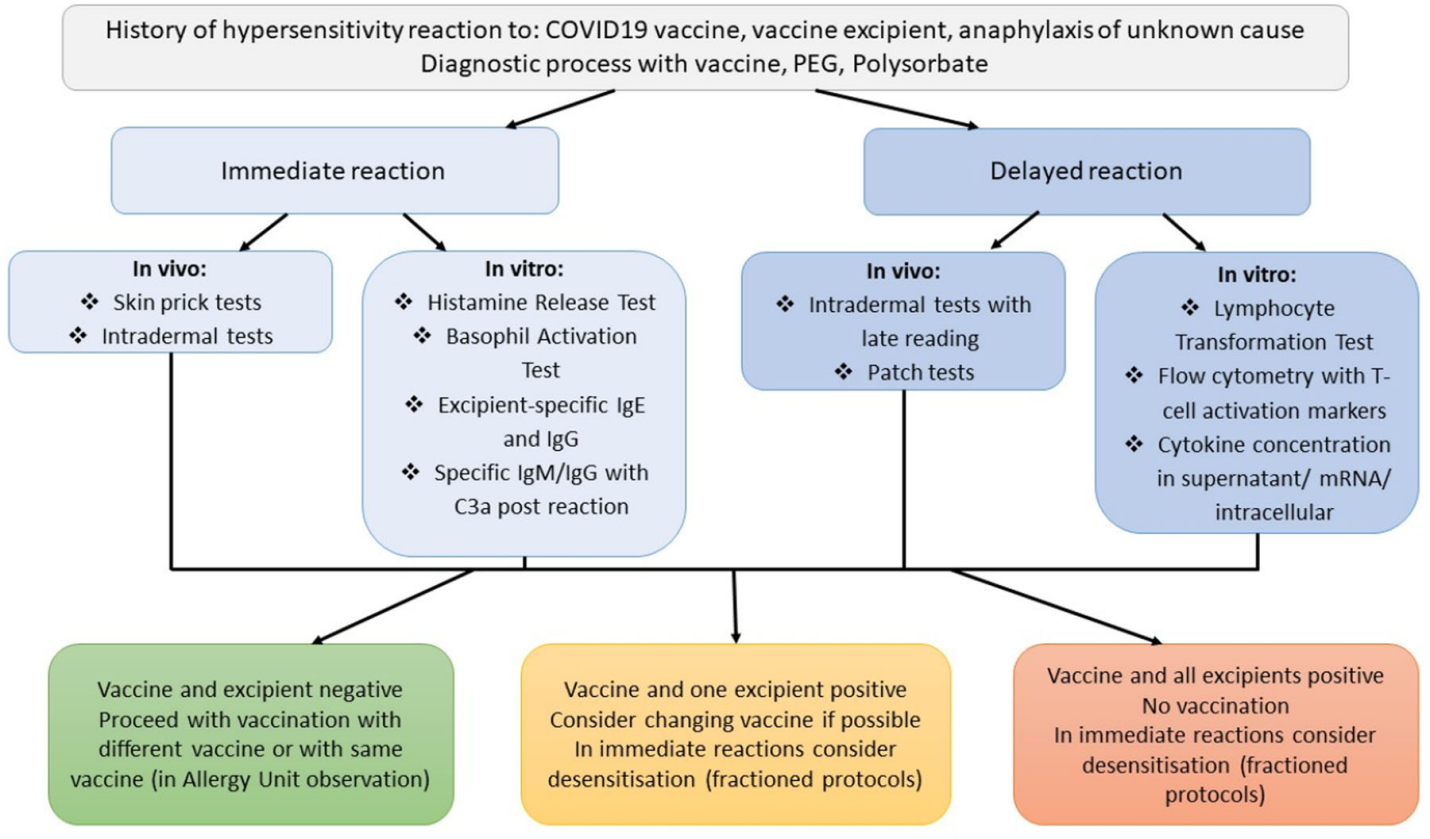
Proposition of allergy work‐up protocol. Adapted from Barbaud et al. and Romantowski et al. with the addition of further In Vitro tests [5, 9].

In the case of delayed reactions, In Vivo tests include: Intradermal tests with late reading (4–6 h postapplication) and patch tests. However, their usefulness in vaccine allergy is debatable due to their probably low sensitivity [9, 36]. In these reactions, LTT and T‐Cell activation markers flow cytometry may possess high utility, but these tests still require more studies. Performing studies with a high number of patients with delayed hypersensitivity to COVID‐19 vaccines might be difficult, firstly, because of the extreme rarity of such reactions (less than 0.1 per 100.000 administrations) [55], and secondly, because the patients may be more reluctant to undergo allergy work‐ups in fear of possible long‐lasting delayed reactions [56]. Proposed test applications are presented in Figure 1.

Finally, it is important to also evaluate other possible allergens that have caused allergic reactions during previous administrations in the injection process, such as latex and chlorhexidine [57].

## 14 Diagnostic Doubts and Difficulties

The hypersensitivity and anaphylactic reactions post COVID‐19 vaccines remain partially unclear. Luc de Chaisemartin et al. showed that some of the patients with immediate reactions do not present specific IgE, IgG or complement activation. The reactions might happen independently of the classic allergy mechanism and elude the above‐presented tests [58]. Further studies in such false‐negative patients are required to understand the reaction pattern and possibly propose further test options.

Secondly, even in patients with a previously confirmed allergy to PEG or pegylated drugs, administrations of a COVID‐19 PEG‐based vaccine might produce no adverse events [59, 60]. The authors suggest that PEG allergy diagnosis is not standardized and might not reflect vaccine exposure. Also, even in the case of confirmed PEG sensitivity, allergic reactions might not be consistent with exposure. Thus, testing with an excipient might provide less value than with the entire COVID‐19 vaccine.

Taking the above into account, cell cultures and In Vitro provocations might be the solution, since they produce a result (effector‐cell activation measurement) regardless of the mechanism. Still, the greatest challenge remains the tested drug concentrations, which require validation in a higher number of subjects. This might be difficult because COVID‐19 vaccine allergy is rare compared to beta‐lactams or NSAIDS [55, 61, 62]. Also, it might be difficult to differentiate a delayed hypersensitivity reaction from proper immune system activation on vaccine exposure since both of these might produce a similar cytokine pattern [36, 63, 64].

## 15 Conclusions

The utility of In Vitro tests increases in the diagnosis of allergy and hypersensitivity to COVID‐19 vaccines, especially in the context of delayed reactions. With their great safety, they present a significant alternative to In Vivo tests considering live provocations. Although BAT and HRT seem promising, still sensitivity of In Vitro tests in immediate type reactions seems low and development might be difficult in the presence of skin tests. Additionally, in cell culture tests and delayed hypersensitivity reactions In Vitro tests require better standardization for appropriate cut‐off points and tested vaccine concentration. Also, differentiation of allergic and normal immune system response remains an issue particularly in cytokine concentration assessment.

## Author Contributions

**Jan Romantowski:** conceptualization, investigation, methodology, resources, visualization, writing – original draft, writing – review and editing. **Marika Gawinowska:** investigation, writing – original draft, writing – review and editing. **Piotr Trzonkowski:** supervision, writing – review and editing. **Marek Niedoszytko:** conceptualization, software, supervision, writing – review and editing.

## Conflicts of Interest

The authors declare no conflicts of interest.

## Data Availability

The authors have nothing to report.
